# Comprehensive influence of topological location and neighbor information on identifying influential nodes in complex networks

**DOI:** 10.1371/journal.pone.0251208

**Published:** 2021-05-21

**Authors:** Xiaohua Wang, Qing Yang, Meizhen Liu, Xiaojian Ma

**Affiliations:** 1 School of Safety Science and Emergency Management, Wuhan University of Technology, Wuhan, China; 2 School of Data and Computer Science, Shandong Women’s University, Jinan, China; 3 School of Management, Wuhan University of Technology, Wuhan, China; University of Burgundy, FRANCE

## Abstract

Identifying the influential nodes of complex networks is now seen as essential for optimizing the network structure or efficiently disseminating information through networks. Most of the available methods determine the spreading capability of nodes based on their topological locations or the neighbor information, the degree of node is usually used to denote the neighbor information, and the k-shell is used to denote the locations of nodes, However, k-shell does not provide enough information about the topological connections and position information of the nodes. In this work, a new hybrid method is proposed to identify highly influential spreaders by not only considering the topological location of the node but also the neighbor information. The percentage of triangle structures is employed to measure both the connections among the neighbor nodes and the location of nodes, the contact distance is also taken into consideration to distinguish the interaction influence by different step neighbors. The comparison between our proposed method and some well-known centralities indicates that the proposed measure is more highly correlated with the real spreading process, Furthermore, another comprehensive experiment shows that the top nodes removed according to the proposed method are relatively quick to destroy the network than other compared semi-local measures. Our results may provide further insights into identifying influential individuals according to the structure of the networks.

## 1 Introduction and motivation

Networks play an important role in people’s social lives nowadays that a wide range of real-world phenomena, from social to medical and biological networks, can be described by complex networks [1, 2]. The nodes play different roles in the network since some nodes are more important than others according to their structural positions. Identification of the important nodes in networks has been a fundamental problem and have theoretical significance in many applications, such as constraining and preventing the spreading of disease [3]or rumor [4] information dissemination [5, 6], developing medicine for illnesses in protein and brain networks [7] and so on.

Many researchers have focused on and stress the problem of identifying the influential nodes [8–11], a node can be influential based on how central the node is to the network, generally, the nodes’ influence in a network can be seen from two aspects [12, 13]. The one from the aspect of the robustness of the network, that is the important nodes are those on which the network structure depends to maintain its connectivity, and the removal of them will cause the whole network to split into the disconnected sub-graphs. The other one from the aspect of spreading, that is to say, nodes within greater spreading capability are regarded as influential ones. Various topology information-based measures have been proposed to identify the important nodes, such as the representative ones like Degree Centrality [14], Betweenness Centrality [15], Closeness Centrality [16], K-shell [17], etc. Usually, these measures can be divided into three types of well-known metrics [18]: local metrics, global metrics, and semi-local metrics. For the first types, the importance of a node is measured according to information of the nearest neighbors. For the second type, the entire graph’s information is needed when evaluating the importance. In recent years, a new classification of measures based on semi-local manners has been developed and can be seen as a trade-off between local and global metrics. Degree centrality [14] is a typical local method with higher degree nodes are considered more influential, and the H-index centrality [19] of a node was extended based on the concept of H-index to identify the spreading capability of nodes. Simplicity and time-efficient are the advantages of these local metrics but they suffer from low accuracy since the more topology structure information is ignored. Closeness centrality [16], Betweenness centrality [15] can be mentioned as typical global ones, these methods focus on the global structure to determine the nodes’influence, while, they lose the efficiency than the local measures in large scale networks since detecting shortest paths between each pair of nodes is time-consuming. Furthermore, Kistak et al. [17] propose the k-shell decomposition method which is time-efficient without losing pays attention to the global location of nodes, a higher Ks value node is considered to be closer to the core of the network and is more influential. However, recent research [20, 21] pointed out the k-shell measure lose its role in the Barabasi–Albert network where the nodes are assigned to the same shell. To rank nodes effectively and efficiently, a semi-local centrality measure [22] has been proposed by taking into more comprehensive neighbor information and shows its accuracy compared with the local metrics and its time efficiency compared with the global metrics.

In addition to the methods mentioned above, some other measures that combine different attributes or different information have been proposed to evaluate the influence of nodes. Considering that the k-shell method ignores the links connecting to the removed nodes, the mixed degree decomposition [20] proposes to combine both the residual degree and the exhausting degree. The neighborhood coreness [23] takes into account the neighbors’ k-shell information. The local structural centrality extends the semi-local centrality measure [24] by taking into account the topological connections among the neighbors. The weight degree centrality method [25] proposes to combine the nodes’ degree and their ability of spreading out. Stating that the node’s influence is not limited up to the nearest neighbors level preferably, the gravity centrality [26] and the local gravity model take into account both neighborhood information and path information to evaluate the node’s influence [27]. The generalized mechanics model enrich it by combining the global information and local information [28]. Considering that the community structure [29] is one common and important structural properties in real-world networks, several measures [30–32] take advantage of the community structure to quantify the influence of nodes, such as, the combination of the number and sizes of communities to which a node directly links [30], and the combination of eintra-community and inter-community links [32].

In general, it has been revealed that the neighborhood attribute and position attribute are two important factors in determining the importance of a node. Inspired by this, this paper proposes a new hybrid centrality to discover these influential nodes. On one hand, the neighbor number is used to denote the neighborhood attribute, and the position attribute is denoted by the proportion of the triangular structures formed by the node and its neighbors. Evaluation results in terms of discriminability, correctness demonstrate that the proposed method can efficiently discriminate the influence capability of nodes and provide a more reasonable ranking list than other compared methods. The remainder of this paper is organized as follows. In Section 1, related work will be reviewed. Section 2 describes the details of the proposed method. Section 3 reports and analyzes the experimental results, followed by a conclusion in Section 4.

## 2 Proposed method

In the current research, it has been attempted to determine the influential nodes using the natural characteristics of networks in a semi-local approach. K-shell is known as the position index of a node in the network, usually, a higher K-shell value means a node is surrounded by large number of denser connected neighbors that the node itself may not be easily removed by every iteration. Once a connection exists between any two of its neighbors, a triangle structure forms. Supposing that many triangle structures formed among the node itself and its neighbors, the node is more likely to locate in a dense part of the network. So, the number of triangles may be an effective indicator in measuring the location of the node, especially, the triangle act as another role, that is, measuring the topological connection among nodes [33], the higher the percentage of the triangular structures formed by a node with its neighbor nodes in the whole network, the denser the connections between the node and its neighbor nodes are. Inspired by this, using the percentage of the triangular structures, we propose a hybrid centrality that considers the neighbor information and position attribute of a node simultaneously. And it is a fact that, during the spreading process, the node usually touches the nearest neighbors first, then the next nearest neighbors, etc. The contact distance between nodes is an important parameter in a spreading process [34], the interaction effect between two nodes decreases with their distance. Unlike any other time-consuming algorithms [35, 36] when calculating the shortest path distance. In this paper, we simplify it as follows, the distance from a node to the nearest neighbors is one, and to the next nearest neighbors is two, etc. In this way, the influence for a node is defined as (labeled as C):

$$C(v)=\sum_{u\in \Phi (v)}\frac{k_{u}*(1+TP(u))}{d^{2}(uv)},$$ (1)

where *k*_*u*_ is the degree of node *u*, *TP*(*u*) is the percentage of triangle structures that exist between the node and its neighborhoods, calculated by: $TP\left(u\right)=\frac{NTS(u)}{TNTS}$, *NTS* is the triangle structures formed between node *u* and its neighborhoods, and *TNTS* is the sum of triangle structures formed by all the nodes in the networks, namely, $TNTS=\sum_{v=1}^{n}NTS\left(u\right)$, the total number of triangle structure exists in the network are $\frac{1}{3}$ *TNTS, and *d*(*uv*) denotes the shortest distance between the node *u* and *v*, the neighborhood set *u* ∈ Φ(*v*) denote the nearby nodes include but not bounded the nearest neighbors, that is to say, more step far away nodes’ information are taken into consideration. To reduce the algorithm complexity, in the paper, the distance ranged *d* is set to be 2, namely, only the nearest neighbors and the next-nearest neighbors are taken into consideration. And the effect of d is validated in Section 3.

Then, an extended index is further developed based on Eq(1), which is defined as (labeled as Lhc):

$$Lhc(v)=\sum_{w\in \tau (v)}C(w),$$ (2)

where *w* ∈ *τ*(*v*) is the nearest neighborhood of node *v*.

The following shows the step of Lhc. The algorithm traverses the nodes in the network in turn. The main work is to calculate the degree value and the number of triangle structures among the node and its neighbor.

**Algorithm 1** Algorithm for our Lhc.

**Input**: network *G* = (*V*, *E*), the total node number *n*;

**Output**: influence capability of each node;

1: **for** *i* = 1 to *n* **do**

2:  Calculate number of triangle structre of node *v*, *NTS*(*v*);

3: **end for**

4: Generate *TNTS* by $TNTS=\sum_{i=1}^{n}NTS\left(v\right)$;

5: Calculate *TP*(*v*);

6: **for** *i* = 1 to *n* **do**

7:  Get *u* ∈ Φ(*v*);

8: **end for**

9: Generate *C*(*v*) using formula (1);

10: **for** *i* = 1 to *n* **do**

11:  Calculate *Lhc*(*v*) using formula (2);

12: **end for**

## 3 Experiment

### 3.1 Dataset

Several real-word networks are chosen in the following discussion, including contiguous states of the United States of America (Contiguous) [37], Dolphins network (Dolphin) [38], Polbook-network (Polbook) [39], Football-network (Football) [40], Jazz musicians network (Jazz) [41], US Air Line (Usair) [42], Co-authorship network of scientists (Netscience) [43], *C*. *elegans* metabolic network (Elegans) [44], the network of international E-road (Euroroad) [45], Western States Power Grid (PowerGrid) [46], the user network of Pretty-Good-Privacy algorithm (PGP) [47] and so on. The basic topological features of these networks are summarized in Table 1. Among which, 〈*k*〉 and *k*_*max*_ denote the average and the maximum degree, 〈*d*〉 denotes the average distance, C and r denote the clustering coefficient [46] and assortative coefficient [48] respectively.

**Table 1:** Some statistical properties of the real networks.

Networks	n	m	k_*max*_	〈*k*〉	〈*d*〉	C	r
Contiguous	49	107	8	4.367	4.163	0.497	0.23340
Dolphin	62	159	12	5.129	3.357	0.259	-0.0436
Polbook	105	441	25	8.4	3.079	0.488	-0.1279
Football	115	613	12	10.66	2.508	0.403	0.1624
Jazz	198	2742	100	27.697	2.235	0.618	0.0202
Usair	332	2126	139	12.807	2.738	0.625	-0.2079
Netscience	379	914	34	4.823	6.04	0.741	-0.0817
Elegans	453	2025	237	8.94	2.664	0.647	-0.2258
Euroroad	1174	1417	10	2.414	18.37	0.017	0.1267
PowerGrid	4941	6594	19	2.669	18.99	0.080	0.0035
PGP	10680	24316	205	4.554	7.486	0.266	0.2382

### 3.2 Evaluation strategies

The effectiveness of the proposed method is empirically evaluated through a series of experiments. The Lhc is compared with other eight well-known measures involving Local, Global and Semi-local metrics from the aspects of discriminability, correctness and robustness. The methods are DC (degree centrality) [14], BC (betweenness centrality) [15], H-index method(H-index) [19], LC (local centrality) [22], *Cnc*_+_(neighborhood coreness) [23], *G*_+_(extended gravity index) [26] and EW(extended weight degree centrality) [25] and LGM(local version of the gravity model) [28].

#### 3.2.1 Discriminability

If nodes have much different influence ability, then the influence capability of nodes can be easily distinguished from each other. In this section, the centrality measures are compared to evaluate how well the discriminability of them. Under the help of Complementary Cumulative Distribution Function (CCDF) [23], we can achieve a clear specification of the ranking distributions of different measures and see the frequency of nodes distribution.

$$CCDF(r)=1-\frac{\sum_{i=1}^{r}n_{i}}{n}$$ (3)

Where *n*_*i*_ denotes the number of nodes with rank *i* on the list, and *n* is the total number of nodes in the network and *r* is the number of ranks. According to the CCDF principle, if *r* → *n*, the discriminability is well and the CCDF plot will slow down; if *r* → 1that means all nodes are assigned in few of ranks and the CCDF plot will decrease rapidly.

**Fig 1:**
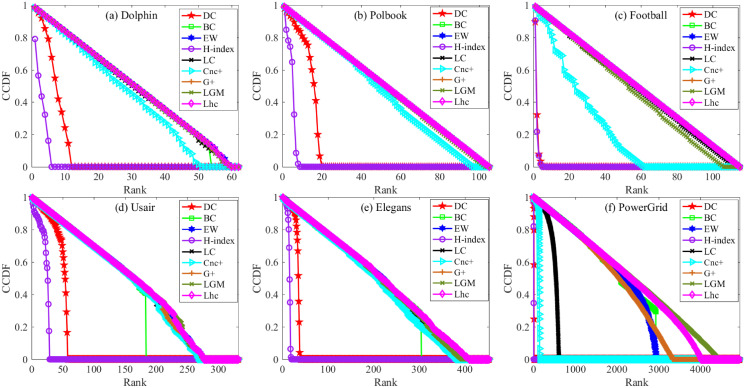
The Complementary Cumulative Distribution Function (CCDF) plot for ranking list offered by different measures. (a)-(d): The CCDF on the network of Dolphin, Polbook, Football, Usair, Elegans and PowerGrid respectively.

The CCDF is plotted for the networks of Dolphin, Polbook, Football, Usair, Elegans, and PowerGrid. As can be seen in Fig 1, In the network of Dolphin, Polbook, and Football, the CCDF of DC and H-index tends to zero with a quick slope, large number of nodes’ influence values cannot be distinguished from each other. The five semi-local methods, LC, EW, *G*_+_, LGM, and our Lhc consider more topological information, so they show better performance, the CCDF plots of them tend to zero with a slower slope following the diagonal line in the network. Though the *Cnc*_+_ consider more neighbors’ K-shell information, the performance seems not to be as well as the LC, *G*_+_, LGM, and Lhc. The BC perform almost the same better performance as LC, EW, *G*_+_ and Lhc, that is to say, the nodes in those three network act as different bridge roles, so a better discriminability the BC method achieve. While, In Usair and Elegans, Lhc tends to show a slower slope and more distinct ranks than LC. As shown in Table 1 about the basic topological statistics of these networks, we can see that the cluster coefficient of the Usair and Elegans network is rather larger, that is to say, a glister of nodes may have many triangle structures formed by the node and its neighbor nodes, our Lhc considers the structure information of a node and its neighbors, so a better ranking distribution it achieves, the CCDF plot of BC slows down at the beginning, then decreases rapidly, that is to say, no more nodes can it distinguish.

When coming to the larger network, PowerGrid, It is clear to note that in the case of DC and H-index, CCDF drops at the beginning like in other networks, BC still cannot achieve a performance as better as the semi-local method even under the circumstance that the BC considers information in the global scope. Particularly, in PowerGrid, the clustering coefficient is small, many nodes encounter with the same degree or K-shell value, so the performance of LC and *Cnc*_+_ are relatively poor compared with Lhc. Lhc shows the best performance even as the fact that EW and *G*_+_ also consider the more step neighbor information of a node. It should be noted that the LGM show better performances than the above methods, the main reason is that the average distance of PowerGrid is large, and more path information are taken into consideration by LGM, so it can achieve better performance in discriminability, but with the expense of time-consuming in this large average distance networks. Nodes in the network may have the same value of H-index, DC even the K-shell value, while the number of triangle structure formed between the node itself and its neighbors may different from each other, so a better ranking distribution performance the Lhc can achieve.

#### 3.2.2 Correctness

Apart from the discriminability evaluation of different measures in the above. In this section, the accuracy and correctness of the proposed measures in node ranking have been evaluated. In principle, the ranked list generated by an effective ranking method should be as consistent as possible with the ranked list generated by the real spreading process. The ranking results of spreading are usually obtained from the SIR model. In the SIR model [17, 49], each node can be in one of three states: susceptible (S), infected (I), and recovered (R). Initially, In detail, to check the spreading influence of one given node, only node *v* is in the infected state, and the other nodes are in the susceptible state. At every time step, each infected node can infect its susceptible neighbors with infection probability *β*, and then it enters into R state with probability *μ*. In this paper, we set *μ* = 1.0. The process continues until no nodes in I state remain in the network. At the end of the SIR process, the number of R nodes is considered as the spreading capability of every node *v*. By selecting different nodes as the initially infected node, the spreading influence of all network nodes and their ranking list can be obtained. In these experiments, the SIR simulation has been repeated 10^4^ times for a network with |*E*| < 100, 10^3^ times for a network with 100 < |*E*| < 1000. The average number of recovered nodes is regarded as their spreading capability. In SIR simulation, the infection probability *β* should neither be too small or too large. When *β* is too small, The epidemic cannot successfully spread over networks, on the contrary, large *β* may lead to an easy outbreak over almost the whole network. So a suitable *β* is needed to better measure the spreading ability of each node. Usually, the value for *β* follows a threshold value, calculated as $\frac{\langle k\rangle }{\langle k^{2}\rangle }$, where 〈*k*〉 and 〈*k*^2^〉 denote the average degrees and average second-order degree of the nodes respectively. The value of *β* is set slightly larger than *β*_*th*_. As Show in Table 2, the *β* for different networks are given.

**Table 2:** The kendall’s tau(*τ*)values between the ranking list obtained from the nine measures and the list offered by the SIR model on eleven networks.

Networks	*β*_*th*_	*β*	*τ*(*DC*, *θ*)	*τ*(*BC*, *θ*)	*τ*(*H*_*in*_, *θ*)	*τ*(*LC*, *θ*)	*τ*(*Cnc*_+_, *θ*)	*τ*(*G*_+_, *θ*)	*τ*(*EW*, *θ*)	*τ*(*LGM*, *θ*)	*τ*(*Lhc*, *θ*)
Contiguous	0.2026	0.21	0.7705	0.5743	0.7282	0.9468	0.9057	0.9371	0.9422	0.8912	**0.9643**
Dolphin	0.147	0.15	0.8130	0.5612	0.7878	0.9251	0.8875	0.9243	0.9180	0.9050	**0.9635**
Polbook	0.0838	0.09	0.7814	0.3669	0.7588	0.9017	0.8987	0.9241	0.9043	0.8099	**0.9266**
Football	0.0932	0.10	0.7151	0.2646	0.5364	0.7970	0.7763	0.7702	0.7802	0.7164	**0.8083**
Jazz	0.026	0.04	0.8722	0.4844	0.8535	0.9477	0.9207	0.9036	0.9402	0.9073	**0.9595**
Usair	0.0225	0.04	0.7638	0.5623	0.7430	0.9053	0.9141	0.9233	0.9160	0.8517	**0.9349**
Netscience	0.1247	0.13	0.6256	0.3956	0.6061	0.8127	0.8392	0.8480	0.8954	0.8059	**0.9036**
Elegans	0.0248	0.03	0.6832	0.4962	0.6038	0.7844	0.8405	**0.8822**	0.8452	0.8061	0.8583
Euroroad	0.333	0.34	0.6037	0.4033	0.5806	0.8888	0.8183	0.8613	0.8468	0.8772	**0.8937**
PowerGrid	0.2583	0.26	0.5899	0.4183	0.4982	0.7814	0.7706	0.7424	0.7417	0.7329	**0.8147**
PGP	0.0553	0.09	0.4772	0.2868	0.7211	0.4553	0.7213	0.7167	0.7414	0.7281	**0.7421**

**Fig 2:**
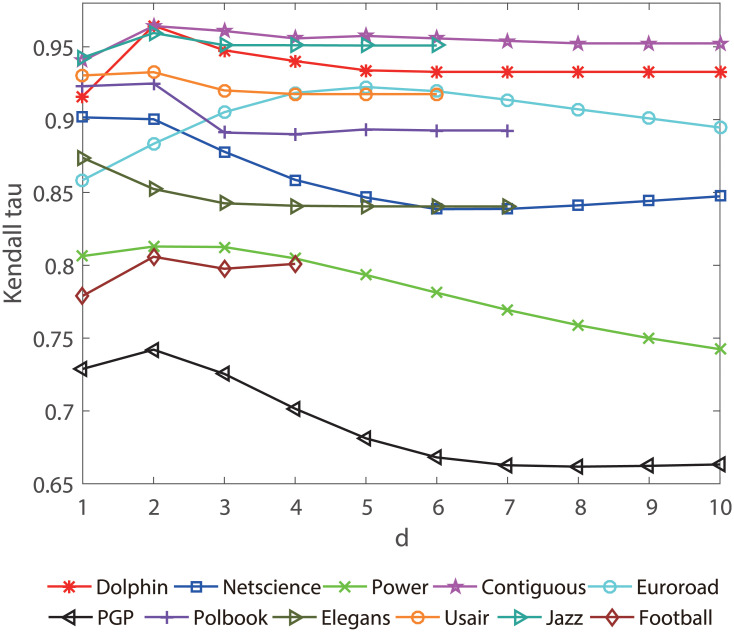
The effects of different parameter *d*. The results are obtained under a series of neighborhood distance range ranges from 1 to 10 on the eleven networks by calculating the kendall’s *τ* between the SIR epidemic ranking list and the Lhc ranking list respectively.

Kendall’s rank correlation coefficientis(*τ*) [50] is usually utilized to quantify the correlation between the ranked list generated by a certain centrality measure and the ranked list obtained from the SIR simulation. Let (*x*_1_, *y*_1_)…(*x*_*n*_, *y*_*n*_) be a set of rank pairs in two distinct ranking list *X* and *Y*. The observations (*x*_*i*_, *y*_*i*_) and (*x*_*j*_, *y*_*j*_) is said to be concordant if *x*_*i*_ > *x*_*j*_ and *y*_*i*_ > *y*_*j*_ or if *x*_*i*_ < *x*_*j*_ and *y*_*i*_ < *y*_*j*_. Otherwise, if *x*_*i*_ > *x*_*j*_ and *y*_*i*_ < *y*_*j*_ or if *x*_*i*_ < *x*_*j*_ and *y*_*i*_ > *y*_*j*_, the pairs is said to be discordant. If *x*_*i*_ = *x*_*j*_ or *y*_*i*_ = *y*_*j*_,the pair is neither concordant nor discordant. Kendall’s tau coefficient (*τ*) is defined as follows:

$$\tau (X,Y)=\frac{N_{c}-N_{d}}{\frac{1}{2}N(N-1)},$$ (4)

where *N*_*c*_ and *N*_*d*_ are the numbers of concordant and discordant pairs in the ranking lists respectively. It is noted that *τ* is positively related to concordant of the ranking lists. A higher *τ* value indicates that the ranked list a centrality measure generated is more correlated to the real spreading process. Previously, in the proposed method, the neighborhood distance range is set by the parameter *d* = 2, that is to say, only the nearest neighbors, next-nearest neighbors are taken into consideration. Under the help of SIR, the effect of different *d* is provided in the following experiment through the ten real networks, including: Contiguous, Dolphin, Polbook, Football, Jazz, Usair, Netscience, Elegans, Euroroad, PowerGrid and PGP. The Kendall *τ* correlation between the SIR epidemic ranking list and Lhc ranking list are obtained under a series of *d*, As shown in Fig 2, in general, the optimal value of *d* is about 2-3. In most cases of the above networks, *d* = 2 shows the higher *τ*,when *d* > 3 or further increased,the *τ* becomes stable.

**Fig 3:**
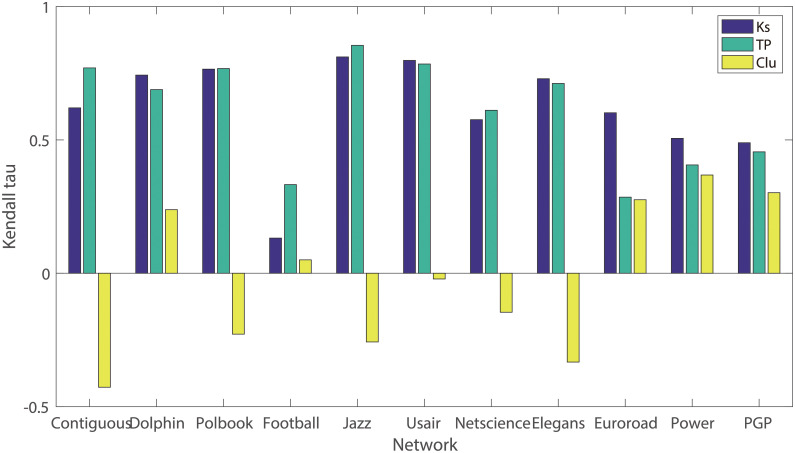
The effects of k-shell(Ks), clustering coefficient(Clu), and the percentage of triangles(TP) on evaluating the spreading ability. The results are obtained by calculating the Kendall’s *τ* between the ranking lists obtained from the three indexes and the list offered by the SIR model on eleven real-world network respectively.

Also, with the help of SIR, the effects of K-shell, Clustering coefficient, and Triangle of nodes on the evaluation of nodes’ influence are compared together. The K-shell value is a known index usually used to measure the location of a node and the Clustering coefficient is usually employed to evaluate the topological connections among the neighbors. While, the triangles, on one hand, can denote the extent that the neighbors may infect each other and on the other hand, it may be an effective indicator in measuring the location of the node. As shown in Fig 3, the clustering coefficient shows its poor performance in evaluating the spreading ability of nodes since the correlation *τ* is rather lower than the other two indexes whether in the denser or sparser network. Sometimes, nodes may have a larger clustering coefficient but relatively fewer triangles, in this case, the effectiveness of the clustering coefficient may not be obvious. Compared with K-shell, the percentage of triangles (TP) shows its comparable performance in the network of which the clustering coefficient are rather higher, and in Contiguous, Polbook, Football, Jazz, and Netscience, Tp achieves better performance than K-shell. While, in some sparser network, such as Euroroad and PowerGrid, of which the connections among nodes are rather smaller, K-shell shows its relatively better performance than TP, that is to say, TP may lose its advantage in this kind of networks, so more topological information is needed, and that is what we proposed Lhc considers and combines: degree and TP, one reflects the neighborhood information of nodes, and the other denotes both the connection among the neighbors and the locations of nodes.

Kendall’s tau correlation coefficients between the two ranking lists for different networks are calculated respectively, the two ranking lists, one is offered by each measure, namely *γ*, where *γ* = DC, BC, H-index, LC, *Cnc*_+_, *G*_+_, EW, LGM, Lhc and the other is obtained from the SIR process, denoted by *θ*. Shown in Table 2, column *τ*(*γ*, *θ*) shows that from small networks like Contiguous, Dolphin to large network like PGP, the *γ* offered by Lhc is highly correlated with *θ* as compared to the other measures.

**Fig 4:**
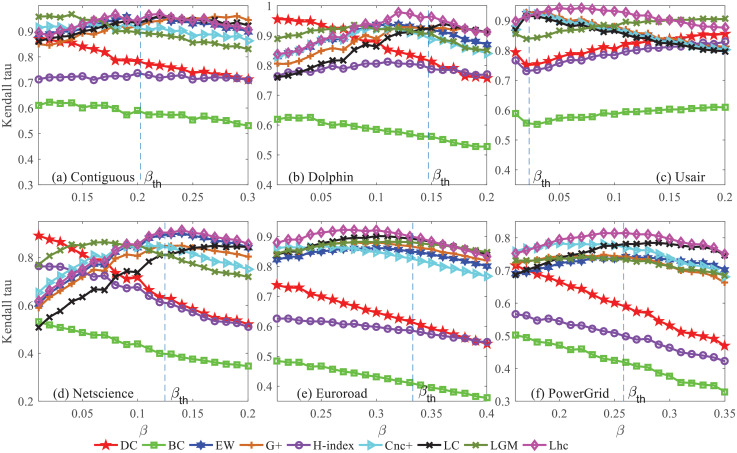
The Kendall’s *τ* between the ranking list from SIR model and that of the eight measures. (a)-(e): The Kendall’s *τ* in the network of Contiguous, Dolphin, Usair, Netscience, Euroroad and PowerGrid respectively. Centrality measures are DC, BC, H-index, LC, *Cnc*_+_, *G*_+_, EW, LGM and Lhc. The dotted line represents the epidemic threshold *β*_*th*_.

To further evaluate how the probability *β* affects the performance of different measures, next, different ranking lists are obtained from the SIR model under a series of *β* which are all around *β*_*th*_. The correlations are plotted for the Contiguous, Dolphin, Usair, Netscience, Euroroad, and PowerGrid networks. As shown in Fig 4, Lhc can achieve better performance with a constant value of the spreading probability *β* in the above networks, especially when *β* is around the epidemic threshold *β*_*th*_, the proposed method is more correlated with *θ*.

In Contiguous and Dolphin, When *β* is far smaller than *β*_*th*_, degree centrality shows its better performance, and as the increase of the spreading probability *β*, the Kendall’s *τ* become lower and lower. Compared with DC and H-index, the six semi-local measures LC, *Cnc*_+_, EW, *G*_+_, LGM, and Lhc perform better as the spreading probability becomes larger to the *β*_*th*_. The larger the spreading probability, the farther away the epidemics can spread from the initially infected node, the LC, *Cnc*_+_, EW, *G*_+_, LGM, and Lhc consider nodes with more steps away from the initially infected node, so they can achieve better performance on a wide range of *β*. The above results confirm the fact that only the local neighbor information is not effective in evaluating the influence of a node. In Usair, of which the clustering coefficient is relatively larger, it means that the connections among nodes are dense, Lhc considers the topological connections structure in evaluating the spreading ability, so a better result it achieves, especially when the *β* is far larger than *β*_*th*_, Lhc still keep its high correctness. In the Netscience network, the DC, and H-index perform better at the beginning, but as the *β* becomes larger, they lose their advantage with the two curves turn to decrease. The clustering coefficient of Netscience is also quite large, so the same reasons can be drawn from Usair why Lhc achieves better performance on a wide range of *β*. As for the two larger networks, Euroroad and PowerGrid. The BC still cannot achieve a better *τ* than other methods, seen in this way, BC is not good at evaluating the spreading influence of nodes in these networks. Different from the above -referred networks with the high average degree and high clustering coefficient, both the average degree and clustering coefficients in Euroroad and PowerGrid networks are relatively small, in other words, the average neighbor number of every node maybe not very much and the topological connections among the nodes may not be that dense. The Lhc achieves better performance when *β* is small, even as the *β* becomes larger, the LC performance almost as well as the Lhc, but Lhc still achieve the largest *τ* when *β* is around *β*_*th*_, the results again certifies its effectiveness and robustness in ranking nodes among the networks with different topological charaeteristics.

**Fig 5:**
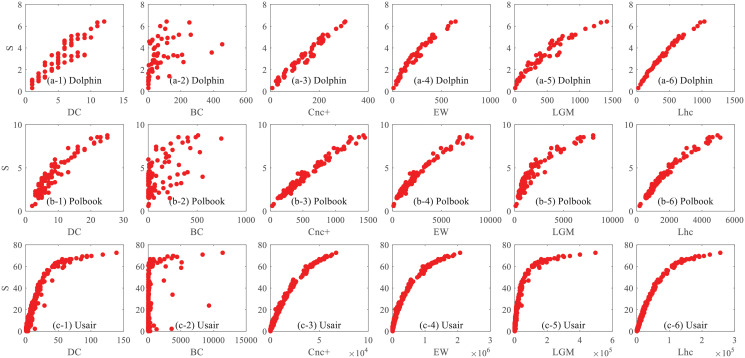
The relationship between node’s influence measured by the SIR model and six centrality measures. From top to bottom, (a), (b) and (c) correspond to the results on Dolphin, Polbook and Usair respectively. Each point indicates a node in the network, the x axis denotes the value of Spreading influence (denoted by S) and the y axis denotes the value of the six centralities respectively, including DC, BC, *Cnc*_+_, EW, LGM and Lhc.

Fig 5 shows the details between the centrality measures and real spreading abilities on three networks, each point indicates a node in the network, the x-axis denotes the centrality value and the y-axis denotes the spreading ability of nodes. In the Dolphin network, both the DC and BC centrality encounter the problem that the spreading ability varies much from each other when the nodes under the same index value. And when comes to the BC centrality, a significant number of nodes are with large spreading influence while the value evaluated by BC is quite small, that is to say, the spreading influence cannot be evaluated by BC properly. The value measured by the centrality method should be consistent with the spreading process, in other words, the larger the centrality value, the better the spreading ability of the node. The *Cnc*_+_, EW, LGM, and Lhc consider more neighbor information, so they perform better than DC and BC, and it can be seen that the real spreading distribution of nodes under the same Lhc value is relatively concentrated. In the Polbook network, the correlation between the value of BC measure and the spreading ability is still not so obvious, and the distribution of spreading ability is relatively scattered when the nodes have the same BC value, especially, some nodes hold larger spreading ability, but their BC value is not necessarily large. The clustering coefficient of Elegans is relatively bigger than other networks, the Lhc takes both the neighbors number and the connections among neighbors into consideration, so a better performance it achieves, and the real spreading distribution of nodes under the Lhc value is relatively more concentrated compared with LC and *Cnc*_+_, the values assigned by Lhc present a more obvious linear relationship with the real spreading.

From the results of the above three networks, we can see that the value of EW, *Cnc*_+_, LGM and Lhc present a positive correlation linear trend with the real spreading ability, that is, the higher the centrality value is, the stronger the node’s spreading ability. However, the correlation between the value evaluated by DC, BC are not that obvious, many nodes hold the same DC index value, but their influence is quite different from each other. Moreover, the performance of DC is not always stable in different networks, the points are concentrated in Usair but are relatively scattered in the Dolphin network. The real influence of a single node shows the good linear correlation with the index value can be well seen in Lhc and compared with other semi-local metrics, Lhc still shows better performance, the influence of multiple nodes assigned with the same Lhc value has little difference, and under the same Lhc value, the real influence distribution of nodes is more concentrated.

**Fig 6:**
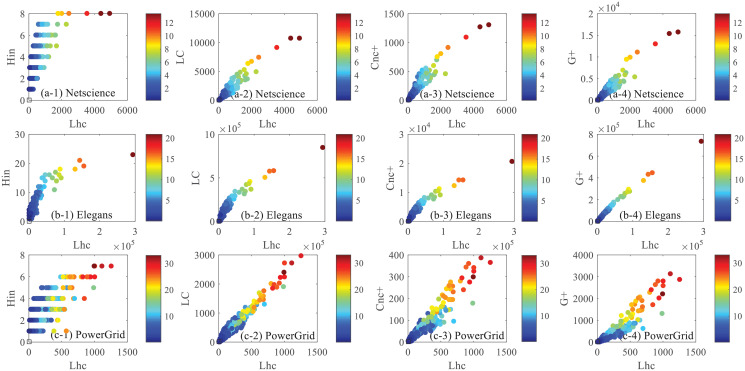
Spreading abilities comparison between Lhc and other four centralities (Hin, LC, *Cnc*_+_, *G*_+_). (a)-(c):The comparison corresponds to the network of Netscience, Elegans and PowerGrid respectively. Each point denotes a node, the x axis denotes the nodes’ Lhc value, and the y axis denotes the value of one of the other four centrality measures. The color represents the spreading influence of this node, namely S.

Fig 6 shows the relations between the Lhc and other four centrality measure on three networks, each point indicates a node in the network, the x-axis denotes the Lhc value and the y-axis denotes the value of the four centrality measures, including the H-index, LC, *Cnc*_+_ and *G*_+_, and the color represents the spreading influence of this node, namely S. In Netscience, the node whose H index is smaller than 7 have no much difference with each other on the spreading influence (with less color variation), while, the spreading influence of the nodes whose H index is 8 have much difference with each other. Seen in this way, H-index may not well evaluate the spreading influence of nodes in Netscience. Comparing with the other three cases, *G*_+_ and Lhc consistent much better with the spreading. In Elegans, the H-index, LC, *Cnc*_+_ and *G*_+_ centralities are all positively correlated with Lhc, especially the *G*_+_ centrality stronger positively correlated relation with Lhc. In addition, we can see that the nodes with higher *G*_+_ centralities and Lhc have deeper color (that is higher influence). In PowerGrid, some nodes have small H-index nodes but higher influence, in the three semi-local methods, the high centrality nodes are likely to have high influence. Compared with H-index, more nearby neighbors’ information is taken into consideration, so the result of LC, *G*_+_ and *Cnc*_+_ consistent much with the spreading. Overall speaking, Among the four cases, the correlation between G+ and Lhc is stronger than the other three cases.

#### 3.2.3 Robustness

**Fig 7:**
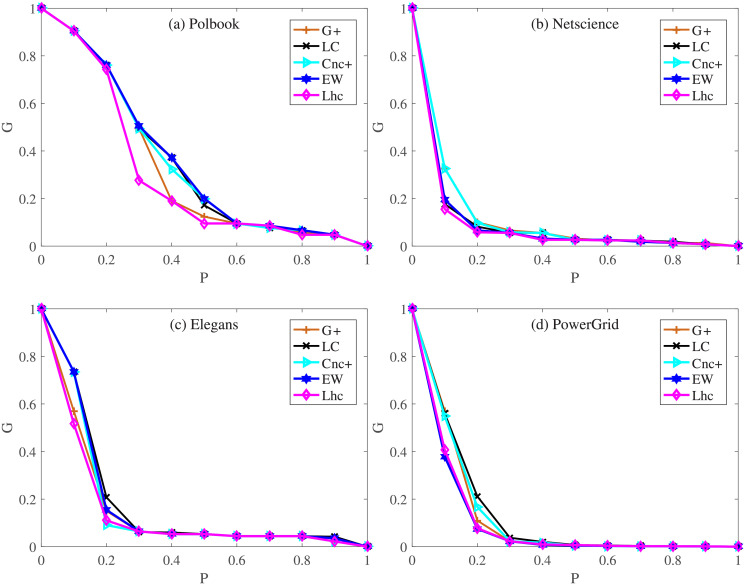
The change of connectivity part after the remove of top influence nodes. (a)-(d): Lhc are compared with other four semi-local methods including LC, *Cnc*_+_, EW and *G*_+_ on the networks of Polbook, Netscience, Elegans and PowerGrid respectively.

In the experiment above, the semi-local manner method have shown their advantages over other local or global methods in evaluating the spreading influence of nodes. Sometimes the whole network can be greatly damaged by attacking a few nodes in the network, in this case, the nodes’ importance lies in the role of maintaining the connectivity of network. In this section, from the perspective of the robustness of the network, the influences of nodes are measured. In the experiment, a certain percentage of nodes in the network are chosen to remove from the network at first, then the change of connectivity part in the network is used to measure the role of the nodes which have been removed before. The ranking of nodes is sorted in descending order by different indexes, and then the nodes with the same proportion (whose value range is [0, 1]) are removed in order. *G* is used to denote the rest giant component of the network after removing the top-k important nodes. The smaller the value of *G*, the more isolated individual nodes or small groups in the network, the more important the removed nodes are. We compare Lhc with other four methods-LC, *Cnc*_+_, EW and *G*_+_ on Polbook, Netscience, Elegans, and PowerGrid. It can be seen from Fig 7, the value of *G* decreases with the number of nodes removed (as the curve decline). In Polbook network, the curve of *G*_+_ and Lhc decline faster than LC and *Cnc*_+_, and Lhc achieves an obvious advantage over other measures after top-30% nodes are removed. In Netscience, removing the top-10% nodes ranked by LC, *G*_+_, Lhc makes the network structure break down quickly and the curve of Lhc is slightly quicker after top-20% nodes are removed. The same conclusions can be drawn from Elegans, the curve of *G*_+_ and Lhc still decline quicker than LC and *Cnc*_+_, especially Lhc performance slightly better after top-10% nodes are removed. The most obvious is the PowerGrid network, the clustering coefficient of PowerGrid network is small, although remove some nodes cannot quickly break down the network structure, top-nodes ranked by Lhc are relatively quick to destroy the network.

## 4 Conclusion

Effectively identify influential nodes in networks is of practical significance in many areas, such as the acceleration of information dissemination and the control of epidemic spreading. In this paper, a hybrid way is adopted by combine two topological structural characteristics of the node to evaluate its influence. The proposed centrality measure considers both the neighbor information and the topological connections information among the neighbor nodes. The neighbor information is reflected by the degree of the node to see how many nodes it connects with and the number of triangles structure centering on the node is utilized to measure how close its neighbors are connected. The interaction influence by different step neighbors is also considered by the fact that the interaction effect between two nodes decreases with their distance. Experimental results conducted on several real-world networks show that the proposed Lhc method is more effective at distinguishes the node’s influence than other conventional centrality methods as well as other semi-local methods. Further, by Kendall’s *τ* correlation coefcient, the rank correlation between the ranked list generated by the SIR model and the different centrality measures are calculated, it shows that the proposed measure outperforms the other methods in evaluating the node’s spreading influence. Finally, the node removal methods are applied to evaluate the effectiveness and performance of the centrality method as well, the result shows that the top nodes ranked according to Lhc are important to the structure of networks since they are relatively quick to destroy the network.

## References

[1] Strogatz SH. Exploring Complex Networks. Nature. 2001;410:268–276. 10.1038/35065725 11258382

[2] Havlin S, Kenett DY, Ben-Jacob E, Bunde A, Cohen R, Hermann H, et al. Challenges in network science: Applications to infrastructures, climate, social systems and economics. Eur Phys J Spec Top. 2012;214(1):273–293. 10.1140/epjst/e2012-01695-x

[3] Lloyd AL, May RM. How viruses spread among computers and people. Science. 2001;292(5520):1316–1317. 10.1126/science.1061076 11360990

[4] Borge HJ, Moreno Y. Absence of influential spreaders in rumor dynamics. Phys Rev E. 2012;85(2):026116. 10.1103/PhysRevE.85.026116 22463288

[5] Medo M, Zhang YC, Zhou T. Adaptive model for recommendation of news. Europhys Lett. 2009;88(3):38005–38010. 10.1209/0295-5075/88/38005

[6] Lü LY, Zhang YC, Yeung C, Zhou T. Leaders in Social Networks, the Delicious Case. PLoS ONE. 2011;6(6):e21202. 10.1371/journal.pone.0021202 21738620 PMC3124485

[7] Lin CY, Chin CH, Wu HH, Chen SH, Ho CW, Ko MT. Hubba: hub objects analyzer—a framework of interactome hubs identification for network biology. Nucleic Acids Research. 2008;36(2):438–443. 10.1093/nar/gkn257 PMC2447731 18503085

[8] Ghalmane Z, Hassouni M EI, Cherifi C. Betweenness centrality for networks with non-overlapping community structure. 2018 IEEE workshop on complexity in engineering. 2018;1–5.

[9] Zhao J, Song YT, Liu F, Deng Y. Hubba: The identification of influential nodes based on structure similarity. Connect Sci.2020;1806203.

[10] Wen T, Pelusi D, Deng Y. Vital spreaders identification in complex networks with multi-local dimension, Knowl-Based Syst.2020;195:105717.

[11] Stephany R, Marinette S, Eric L, Hocine C. Interplay Between Hierarchy and Centrality in Complex Networks. IEEE Access.2020;8:129717–129742. 10.1109/ACCESS.2020.3009525

[12] Borgatti SP. Identifying sets of key players in a social network. Comput Math Organiz Theor. 2006;12(1):21–34. 10.1007/s10588-006-7084-x

[13] Zareie A, Sheikhahmadi A. A hierarchical approach for influential node ranking in complex social networks. Expert Syst Appl.2006;93:200–211.

[14] Freeman LC. Centrality in social networks conceptual clarification. Soc Networks. 1978;1(3):215–239. 10.1016/0378-8733(78)90021-7

[15] Freeman LC. A set of measures of centrality based on betweenness. Sociometry. 1977;40(1):35–41. 10.2307/3033543

[16] Sabidussi G. The centrality index of a graph. Psychometrika. 1966;31(4):581–603. 10.1007/BF02289527 5232444

[17] Kitsak M, Gallos LK, Havlin S, Liljeros F, Muchnik L, Stanley HE, et al. Identification of influential spreaders in complex networks. Nat Phys. 2010;6(11):888–893. 10.1038/nphys1746

[18] Lü LY, Chen DB, Ren XL, Zhang QM, Zhang YC, Zhou T. Vital nodes identification in complex networks. Physics Reports. 2016;650:1–63. 10.1016/j.physrep.2016.06.007

[19] Lü LY, Zhou T, Zhang QM, Stanley HE. The H-index of a network node and its relation to degree and coreness. Nature Communications. 2016;7:10168. 10.1038/ncomms10168 PMC4729922 26754161

[20] Zeng A, Zhang CJ. Ranking spreaders by decomposing complex networks. Phys Lett A. 2013;377(14):1031–1035. 10.1016/j.physleta.2013.02.039

[21] Wang ZX, Zhao Y, Xi JK, Du CJ. Fast ranking influential nodes in complex networks using a k-shell iteration factor. Physica A. 2016;461:171–181. 10.1016/j.physa.2016.05.048

[22] Chen DB, Lü LY, Shang MS, Zhang YC, Zhou T. Identifying influential nodes in complex networks. Physica A. 2011;391(4):1777–1787.

[23] Bae J, Kim S. Identifying and ranking influential spreaders in complex networks by neighborhood coreness. Physica A. 2014;395(4):549–559.

[24] Gao S, Ma J, Chen ZM, Wang GH, Xing CM. Ranking the spreading ability of nodes in complex networks based on local structure. Physica A. 2014;403(6):130–147.

[25] Liu Y, Wei B, Du YX, Xiao FY, Deng Y. Identifying influential spreaders by weight degree centrality in complex networks. Chaos Soliton Farct. 2016;86:1–7. 10.1016/j.chaos.2016.01.030

[26] Ma LL, Ma C, Zhang HF, Wang BH. Identifying influential spreaders in complex networks based on gravity formula. Physica A. 2016;451:205–212. 10.1016/j.physa.2015.12.162

[27] Li Z, Ren T, Ma XQ, Liu SM, Zhou T. Identifying influential spreaders by gravity model. Sci Rep.2019;9(1):8387. 10.1038/s41598-019-44930-9 31182773 PMC6557850

[28] Liu F, Wang Z, Deng Y. GMM: A generalized mechanics model for identifying the importance of nodes in complex networks. Knowl-Based Syst.2020;193:105464. 10.1016/j.knosys.2019.105464

[29] Cantwell G T, Newman M E. Mixing patterns and individual differences in networks. Phys Rev E.2019;99(4):042306. 10.1103/PhysRevE.99.042306 31108687

[30] Zhao ZY, Wang XF, Zhang W, Zhu ZL. A Community-Based Approach to Identifying Influential Spreaders. Entropy.2015;17:2228–2252. 10.3390/e17042228

[31] Ghalmane Z, Elhassouni M, Cherifi C, Cherifi H. Centrality in modular networks. EPJ Data Sci.2019;8(15).

[32] Ghalmane Z, Cherifi C, Cherifi H, Hassouni ME. Centrality in Complex Networks with Overlapping Community Structure. Sci Rep.2019;9:10133. 10.1038/s41598-019-46507-y 31300702 PMC6626036

[33] Han ZM, Chen Y, Li MQ, Liu W, Yang WJ. An efficient node influence metric based on triangle in complex networks. Acta Phys Sin-Ch Ed. 2016;65:168901.

[34] Keeling MJ, Eames KTD. Networks and epidemic models. J R Soc Interface. 2005;2(4):295–307. 10.1098/rsif.2005.0051 16849187 PMC1578276

[35] Dijkstra EW. A note on two problems in connexion with graphs. Numer Math. 1959;1(1):269–271. 10.1007/BF01386390

[36] Floyd RW. Algorithm 97: Shortest Path. Comm Acm. 1962;5:345. 10.1145/367766.368168

[37] Contiguous USA network dataset—KONECT; 2017. http://konect.uni-koblenz.de/networks/contiguous-usa.

[38] Lusseau D, Schneider K, Boisseau OJ, Haase P, Slooten E, Dawson SM. The bottlenose dolphin community of Doubtful Sound features a large proportion of long-lasting associations. Behav Ecol Sociobiol. 2003;54(4):396–405. 10.1007/s00265-003-0651-y

[39] Krebs V. USPolbooks;. http://www.orgnet.com.

[40] Girvan M, Newman M E. Community structure in social and biological networks. Pans. 2002;99(12):7281–7286. 10.1073/pnas.122653799 PMC122977 12060727

[41] Gleiser PM, Danon L. Comunity structure in jazz. Adv Complex Syst. 2003;06(04):565–73. 10.1142/S0219525903001067

[42] Batagelj V, Mrvar A. Usair. http://vlado.fmf.uni-lj.si/pub/networks/data/.

[43] Newman ME. Finding community structure in networks using the eigenvectors of matrices. Phys Rev E.2006;74(32):036104. 10.1103/PhysRevE.74.036104 17025705

[44] Jordi D, Alex A. Community detection in complex networks using extremal optimization. Phys Rev E. 2005;72(2):027104. 10.1103/PhysRevE.72.027104 16196754

[45] Šubelj L, Bajec M. Robust Network Community Detection Using Balanced Propagation. Eur Phys J B. 2011;81(3):353–362. 10.1140/epjb/e2011-10979-2

[46] Watts DJ. Collective dynamics of ‘small-world’ networks. Nature. 1998;393:440–442. 10.1038/30918 9623998

[47] Marián Boguñá, Romualdo Pastor-Satorras, Albert Díaz-Guilera, Alex Arenas. Models of social networks based on social distance attachment. Phys Rev E. 2004;70:056122. 10.1103/PhysRevE.70.056122 15600707

[48] Newman ME. Assortative mixing in networks. Phys Rev Lett. 2002;89:208701. 10.1103/PhysRevLett.89.208701 12443515

[49] Moreno Y, Pastor-Satorras R, Vespignani A. Epidemic outbreaks in complex heterogeneous networks. Eur Phys J B. 2002;26(4):521–529.

[50] Kendall MG. A new measure of rank correlation. Biometrika. 1938;30(1/2):81–93. 10.2307/2332226

